# Life in a New Normal with a Self-Care Routine: A Cross-Sectional Study of Older Adults’ Daily Health Behaviors (DHB) Performance during the Initial Outbreak of COVID-19 in China

**DOI:** 10.3390/nu14081678

**Published:** 2022-04-18

**Authors:** Xiaoyuan Jin, Ying Chen, Rui Zhou, Xiaole Jiang, Boyan Chen, Hao Chen, Ying Li, Zhi Chen, Haihong Zhu, Hongmei Wang

**Affiliations:** 1 Department of Social Medicine of School of Public Health and Department of Pharmacy of the First Affiliated Hospital, Zhejiang University School of Medicine, Hangzhou 310058, China; xiaoyuanj@zju.edu.cn (X.J.); chenying1996@zju.edu.cn (Y.C.); zhou_rui@zju.edu.cn (R.Z.); 3160105297@zju.edu.cn (X.J.); chen_b_y@zju.edu.cn (B.C.); chen_hao97@zju.edu.cn (H.C.); 2 Department of Public Health, Xi’an Medical University, Xi’an 710021, China; ly448656465@163.com; 3 State Key Laboratory for Diagnosis and Treatment of Infectious Diseases, National Clinical Research Center for Infectious Diseases, National Medical Center for Infectious Diseases, Collaborative Innovation Center for Diagnosis and Treatment of Infectious Diseases, The First Affiliated Hospital, Zhejiang University School of Medicine, Hangzhou 310003, China; zjuchenzhi@zju.edu.cn (Z.C.); zhuhh72@zju.edu.cn (H.Z.)

**Keywords:** self-care, daily health behaviors (DHB), Coronavirus disease 2019 (COVID-19), older Chinese

## Abstract

For older adults, self-care begins with daily health behaviors (DHB), which refers to a series of basic behaviors beneficial to health in daily life; it is the foundation for promoting health, preventing disease, and maintaining health with or without the support of a healthcare provider. Thus, this study aimed to observe the changes in DHB among older adults when the COVID-19 pan-demic first erupted in China (at the beginning of 2020) and explore the impact factors on self-care routines in daily life. We applied a cross-sectional study among 1256 (83.7%) valid older Chinese from 19 February 2020 to 19 March 2020, the score of DHB changes (mean ± SD, 14.70 ± 2.140; range, 8–18) presented a significant growth (t_1256_ = 44.636, *p* < 0.001) during COVID-19. From 3 hierarchical linear regression models, the older Chinese who received a higher education include high school (*β* = 0.403, 95% CI [0.009, 0.797], *p* = 0.045) and college degree and above (*β* = 0.488, 95% CI [0.034, 0.943], *p* = 0.035), and lived in the eastern China (*β* = 0.771, 95% CI [0.392, 1.151], *p* < 0.001) took DHB more frequently. However, the high-risk infection (*β* = −0.740, 95% CI [−1.248, −0.231], *p* = 0.004), overweight/obese character (*β* = −0.265, 95% CI [−0.526, −0.004], *p* = 0.047), and alcohol consumption (*β* = −0.350, 95% CI [−0.634, −0.065], *p* = 0.016) are significant factors in decreasing a senior’s DHB performance. For China, self-care offers a straightforward strategy among the range of measures required to combat COVID-19 and future health threats. In summary, findings in this study can build a foundation for developing healthcare policy and services for the relevant government and departments on prompting DHB and the importance of self-care among the older population.

## 1. Introduction

In December 2019, the first known case of severe acute respiratory syndrome, coronavirus 2 (SARS-CoV-2), emerged from Wuhan, China [1]; the term COVID-19 (Coronavirus disease 2019) was developed and became defined as a worldwide pandemic [2]. To date, COVID-19 has remained an imminent, severe, and global health emergency public’s cause for concern. Specifically, the non-fully developed clinical treatment with unknown viral characteristics [3,4], low acceptance of vaccination by the population [5,6], and the continued emergence of new viral variants [7,8] burden all countries and governments’ various pressures. Except for the barriers to infectious control, the uncertainties also include significant global variability of mortality due to COVID-19 [1,4,7]. An observational study conducted in the U.S. found an excess of 122,300 (95% prediction interval: [116,800~127,000]), more deaths than would typically be expected at the same time of the year [8]. Except for mortality cases due to the severe pandemic, data also recorded indirect deaths caused by reduced immune function [8,9,10,11] and cognitive impairment (for example, cardiovascular events, delayed cancer care, or malnutrition) [8,12,13,14]. Frail and subject to multimorbidity, older adults are at the highest risk for severe and fatal diseases [10,13,15]. Several epidemiological data have indicated that the prevalence of COVID-19 is more elevated in older than younger persons [11,12]. In short, the global mobilization to fight the health consequences of COVID-19 has been impressive but more work is needed. As these alarming trends continue, engaging in self-care routines is among the most promising and exciting new approaches to improving health and well-being [16,17,18].

Given the authorized definition from the World Health Organization (WHO), “self-care is the ability of individuals, families, and communities to promote health, prevent disease, maintain health, and to cope with illness and disability with or without the support of a healthcare provider” [19,20]. In this definition, the scope of self-care includes health promotion, disease prevention and control [17,21]; lifestyle routines for care-dependent persons [22,23]; and rehabilitation, including palliative care [24,25]. However, the current infection controls relied more on preventive behaviors (for example, wearing masks regularly, taking vaccines) [26,27], but the efforts on prompting the lifestyle routines were not enough, especially for older adults who inherently require healthy lifestyle habits. Self-care begins with daily health behaviors (DHB) to keep both the self-body and the self-mind fit and healthy [20,28]. Specifically, the DHB refers to a series of basic behaviors beneficial to health in daily life (for example, maintaining personal hygiene, reaching adequate sleep, eating reasonable nutrition, doing regular exercise, and taking appropriate medications for minor illnesses) [22,28].

According to the practice guidelines published in 2019 and 2021 by WHO [20,29], the self-care interventions, if situated in an environment that is safe and supportive, constitute an opportunity to help increase people’s active participation in their health, including the subject’s engagement. However, given the concerns about the increasing spread of COVID-19, infection control has altered life patterns among the general public, causing changes in people’s lifestyle behaviors [30,31]. Existing studies also indicated that mandatory isolation measures are expected to decrease the adoption of healthy dietary behaviors [32,33,34,35] and physical activities [18,33,36], which caused unforeseen medium- and long-term consequences for mental and physical health [18,31,37].

Therefore, using a self-administrated questionnaire, this paper aims to assess changes in the use of DHB among older people during the initial COVID-19 outbreak in China and explore the influential factors. Other questions from the self-designed survey evaluated the older Chinese’s perceived attitude, preventive behaviors, and mental health status, which have already been published elsewhere [38,39]. Since the same survey was applied, all the papers shared and presented a standard method description.

## 2. Materials and Methods

This research worked during the initial breakdown of COVID-19 in China, which had taken strict control of the disease with national lockdown and self-isolation at home [14,26]. We conducted an online survey to collect data. During the study period, a Big-data technology, Health QR Code (which works to follow Chinese residents’ real-time health status based on their current trajectory), supplemented researchers to recruit healthy participants to meet the study’s basic certifications.

### 2.1. Study and Population Design 

From 19 February 2020 to 19 March 2020, the study applied a convenience sampling method to conduct an online self-administrated survey in simplified Chinese (uploaded on the Web platform “*Wenjuanxing*”), specifically by distributing the shared web link or scanned QR code on WeChat (the most popular social media in China). In total, 1501 respondents were collected from 31 provinces of mainland China. The study involved 1256 responses (83.7%) in data analysis without invalid questionnaires with incomplete information and logical error. 

The eligibility criteria required all older Chinese (≥60 years) without intellectual or cognitive impairment and those willing to participate in the study. To minimize the incomplete questionnaires, we attached research assistants’ contact information to provide phone-call help and remote guides on solving technique issues during the answering process. In the case of surrogate completion, it was emphasized that the answers must reflect the true thoughts of the respondents. The study rewarded respondents who completed the questionnaire with a monetary incentive of RMB 1–5 (the U.S. $0.15–0.76) through the online prize wheel. All of the respondents were not informed of monetary incentives before starting the questionnaire.

### 2.2. Measurements

This paper presents preliminary data on self-rated changes in the frequency of DHB among older Chinese before and during home confinement due to the COVID-19 outbreak. Before the formal survey, a pilot study was conducted with 30–40 older Chinese to test the feasibility of the questionnaire, and the results showed that the self-designed questions were acceptable for respondents. 

#### 2.2.1. Outcome Variable

The outcome variable is the changes in DHB among older Chinese during the COVID-19 pandemic, which were measured by a self-administered questionnaire. All behavior-related questions were designed on the WHO’s practical guidelines on self-care interventions for health [20]. Specifically, the measurement was designed to ask participants’ self-assessed frequency changes in their health behaviors using the phrase, “*How often did you [particular DHB category] in the past week?*”. Based on the self-care pillars [22]: hygiene (general and personal), nutrition (type and quality of food eaten), lifestyle (sporting activities, leisure, etc.), and self-medication, the evaluated DHB included 6 self-care activities—(1) Opening the door/window to keep interactive ventilation; (2) Washing hands; (3) Performing physical activities; (4) Eating vegetables and fruits (V.F.), and rich-protein diets; (5) Taking vitamins/medical supplements; and (6) Having a high-quality sleep with enough hours. The assessed results were shown as the changes in taking frequency, with response options of ‘decreased’, ‘no changes’, and ‘increased’.

#### 2.2.2. Covariates 

The covariates are composed of several questions regarding participants’ socio-demographic characteristics, physical well-being, and health self-assessment. In detail, data on sex, age, marital status, educational level, registered residential area (stratified into ‘*rural*’ and ‘*urban*’), socio-economic status (SES), and the living environment were collected as socio-demographic characteristics. Considering all eligible participants reached the legal retirement age in China (60 years for male workers, 55 years for female cadres, and 50 years for female workers [40]), we asked SES from retired types (‘*semi-retirement*’, ‘*retirement with honors*’, and ‘*traditional retirement*’) [41] and monthly earning household income (RMB). In addition, we evaluated participants living environment during the survey period by collecting their self-reported living regions (classified into different geographic areas in mainland China as ‘*eastern*’, ‘*central*’ and ‘*western*’ regions) and categorizing the risk level of COVID-19 according to the authorized data from the National Health Commission (accessed on 20 March 2020) [42] (classified cumulative confirmed cases < 100, 100–999, and ≥1000 as ‘*low-risk*’, ‘*medium-risk*’, and ‘*high-risk*’ areas, respectively).

The questionnaires related to physical well-being collected respondents’ health information from body mass index (BMI) categories (calculated BMI value from self-reported weight in kilograms divided by the height in meters squared, and classified the respondents as ‘*underweight (<18.5 kg/m^2^)*’, ‘*normal weight (18.5~23.9 kg/m^2^)*’, and ‘*overweight/obesity (≥24.0 kg/m^2^)*’ [43]); habits on smoking and alcohol consumption, which forced the question participants’ current actions of doing or not; and the status of the chronic diseases (calculated number of diagnosed chronic diseases respondents circled from the responding list, and classified participants into ‘*no chronic disease*’, ‘*1–2 chronic disease(s)*’, and ‘*multiple (3 or more) chronic diseases*). The last domain of the variables includes self-assessment of health, classified and measured into ‘*Poor*’, ‘*Fair*’, ‘*Good*’, and ‘*Very Good/Excellent*.

### 2.3. Statistical Analysis 

The descriptive study summarized and presented data by frequencies, percentages, and mean ± standard deviation (SD). The data analysis graded each DHB category’s frequency changes in ‘*decreased*’, ‘*no change*’, and ‘*increased*’ as points 1, 2, and 3, respectively, and calculated the total score of overall DHB by adding scores of each particular behaviors, which ranged between 8 and 18 among 1256 participants. On the overall DHB, we calculated a one-sample *t*-test and assumed the score of 12 presents no change in overall DHB performance in the past week. Independent *t*-tests and one-way analysis of variance (ANOVA) were used to determine differences between strata groups under each covariate. The hierarchical linear regression model was applied to explore the multivariable analysis of DHB in older adults. The modeling process involved variables related to socio-demographic characteristics, physical well-being, and self-assessment of health into three blocks. Data management and analysis were performed using SPSS 26.0. The significance level was set at a *p* < 0.05.

### 2.4. Ethics Statement

The Zhejiang University School of Public Health ethics committee reviewed and approved the study protocol (ZGL202002-2). The beginning of the questionnaire provided an announced paragraph to respondents that submitting the questionnaire is considered informed consent. Subjects participated in the study anonymously and voluntarily. 

## 3. Results

### 3.1. Participants’ Characteristics

The general information of the total study sample (*n* = 1256; 55.2% were female and 49% aged 60 to 69 years) are reported in Table 1. In detail, 45.5% (*n* = 572) older Chinese received primary school education and below; 74.7% (*n* = 938) were married or cohabiting; and 53.9% (*n* = 677) registered as rural residents. The majority of respondents (*n* = 1047, 83.4%) retired with the traditional option, and over half of those questionnaire (*n* = 842, 67%) reported their monthly household income between RMB 600–6000 Yuan. During the survey period, territorial coverage spread over all regions of mainland China: 49.5% (*n* = 622) lived in eastern China, 15.2% (*n* = 191) in central China, and 35.3% (*n* = 443) in western China; but nearly half of the subjects (*n* = 594, 47.3%) lived with a medium risk level of COVID-19. When asked about participants’ physical well-being, approximately three-fifths (*n* = 749, 59.6%) had normal weight with an average BMI value, most older adults had never or already quit smoking habits (*n* = 1018, 81.1%) and alcohol consumption (*n* = 852, 67.8%), and only 15.8% (*n* = 198) reported with 3 or more chronic diseases. However, only 22.1% (*n* = 277) of the participants self-rated their health status as very good/excellent.

### 3.2. Changes in Each Evaluated DHB during the COVID-19 Pandemic

As shown in Table 2, most respondents self-reported increased the times to open doors and windows (*n* = 947, 75.4%) and wash hands (*n* = 1018, 81.1%) as the 2 most common self-care activities during the pandemic. While these 2 behaviors could not be the direct evidence to present changes in DHB, the increased engagements above could be interpreted as the efficient promotion of infection-prevent measures the other way. Regrettably, the DHB of physical activities was the highest percentage of the decreased frequency (*n* = 357, 28.4%), which may be impacted by the pandemic-imposed restrictions implemented during the survey period. Our previous publication applied the same study population also proved it—1050 respondents met ‘*Entry/exit control exercised*,’ and 181 answered with ‘*lockdown*,’ which limited room to perform physical activities [39]. Among 1256 older adults, Table 2 reveals the apparent prevalence of increasing other behaviors: 53.4% (*n* = 671) improved access to eating healthy diets, including various fruits, vegetables, and rich protein foods; 28.2% (*n* = 354) increased times on taking vitamins or medical supplements, and 47.3% (*n* = 594) self-reported with more high-quality sleep.

In summary, the participants reported a positive frequency change in various self-care activities in the past week, but with a slight increase. In particular, many participants also self-reported with no changes in healthy diets (*n* = 523, 41.6%), self-medication (*n* = 803, 63.9%), and sleep (*n* = 598, 47.6%). These changes may continue to adversely impact the health of older adults as the pandemic persists; thus, only analyzing the impact factors on each specific behavior is not enough. Instead, the more efficient approach will be exploring the motivating factors of overall changes in DHB that would prompt the older adults to perform self-care activities directly or indirectly. 

### 3.3. Total Scores on Changes in DHB among Older Chinese

Among 1256 participants, the total score of their reported changes on 6 DHB categories (mean ± SD, 14.70 ± 2.140) presented an increased taken frequency significantly in the past week (t_1256_ = 44.636, *p* < 0.001) (Table 2), and Table 1 presented participants under each level of observed variables self-reported DHB performance with an increased frequency (the mean score for all subgroups is above 12). From these, the socio-demographic characteristics of age, marital status, education level, registered residential area, and monthly household income are statistically associated with increased DHB during the COVID-19 pandemic (*p* < 0.05) (Table 1). In detail, seniors aged between 60 and 79 years (vs. ≥80 years, *p* = 0.003), married or cohabiting (vs. unmarried/divorced/separated/widowed seniors, *p* = 0.001), registered as urban residences (vs. rural residences, *p* < 0.001), and earning a higher monthly income (>6000 RMB) (*p* = 0.012) are more likely to access DHB frequently. Compared with respondents who lived in western China during the survey period, those in the eastern and central regions were more likely to intake DHB (*p* < 0.001). However, the results revealed that seniors who reported an elementary school education level or below had an indistinctive increased frequency on DHB performance (*p* < 0.001) compared to those who answered with higher education (including middle school, high school, and college or above). Moreover, there was no significant association between the multiple risk levels of COVID-19 in the living regions of the participants and their changes in DHB during the survey period.

As shown in Table 1, participants’ physical well-being (including BMI category, smoking and alcohol consumption habits, and the number of diagnosed chronic diseases) and their self-rated health status also had significant effects on their increased DHB. In particular, older adults who were normal weight (vs. the underweight and overweight/obese groups, *p* = 0.022) were more likely to access DHB with increased frequency. The older adults who never or have already quit smoking (*p* = 0.023) and drinking (*p* = 0.016) presented significant effects on their increased frequency of DHB during COVID-19. Furthermore, the results showed a significant difference between the different numbers of chronic diseases: Seniors diagnosed with three or more chronic diseases were the most suboptimal group in implementing DHB, compared to those without chronic diseases or those who only have 1–2 types *(p* = 0.041). Finally, participants who self-rated their health status as ‘*Poor*’ were less likely to take up various DHB frequently during COVID-19.

### 3.4. Hierarchical Linear Regression Model 

The multivariable analysis of the total score of changes in various DHBs applied three hierarchical linear regression models, including all the covariates from the whole study population. In detail, participants’ socio-demographic characteristics, also including the evaluations of their socio-economic status and living environment, were entered into Block 1, accounting for a variance of 4.7% (R^2^ = 0.047, F = 3.808, *p* < 0.001). Block 2 involved variables related to the group sample’s physical well-being, including BMI categories, habits of smoking and drinking, and the number of chronic diseases to explain the variance of 1.1% (R^2^ = 0.058, F = 3.453, *p* < 0.001). In addition, Block 3 was built with the parameters of self-rated health status, but the result did not present more variance (R^2^ = 0.059, F = 3.111, *p* < 0.001) (Supplementary Table S1). Therefore, the results presented that education level, living regions, the current risk level of COVID-19, BMI categories, and drinking habits have significant effects on DHB among older Chinese during the COVID-19 pandemic (Table 3).

In summary, compared to the educational level of older adults in primary school and below, those who received higher education from high school (*β* = 0.403, 95% CI [0.009, 0.797], *p* = 0.045) and a university degree and above (*β* = 0.488, 95% CI [0.034, 0.943], *p* = 0.035) are more frequently access DHB during the initial breakout of COVID-19. During the survey period, participants who lived in eastern China (vs. western China, *β* = 0.771, 95% CI [0.392, 1.151], *p* < 0.001) significantly increased taking DHB, but those who lived with a high-risk level of COVID-19 took DHB without a remarkable increase (*β* = −0.740, 95% CI [−1.248, −0.231], *p* = 0.004). After adjusting for variables of sociodemographic characteristics and physical well-being, the older adults who are overweight or obese (vs. normal weight, *β* = −0.265, 95% CI [−0.526, −0.004], *p* = 0.047), and those who have habits of alcohol consumption (vs. never/have quit drinking, *β* = −0.350, 95% CI [−0.634, −0.065], *p* = 0.016) reported increasing DHB statically nonsignificant during COVID-19.

## 4. Discussion

The COVID-19 pandemic has sparked interest in health at the individual and community level. To date, it is clear that the management of health risks arising from infectious and chronic diseases necessarily involves informed and supported actions by individuals, such as self-care activities. Thus, everyone is encouraged to adopt DHB and practices to move to a ‘new normal’ as self-care routines during COVID-19. Especially for the elderly, as a susceptible population, maintaining good habits of DHB is essential to practice self-care skills and then maintain personal well-being [23,45]. This study aimed to evaluate self-care activities among older adults during COVID-19 in China and explore the factors that motivate them to have self-care routines. 

### 4.1. Associated Factors on DHB among Older Chinese

#### 4.1.1. Socio-Demographic Characteristics

There is compelling national and international evidence that self-care routines are affected by socio-demographic factors [39,46,47]. The results supported the previous findings and indicated that elementary-level and lesser educated seniors reported an indistinctive increased change in DHB during the pandemic. A similar study by Hu et al. [48] found that subjects with higher educational levels performed more healthy lifestyle behaviors. Not only the studies about COVID-19 but an existing finding from older patients with chronic diseases also summarized that those who had higher education performed better health behaviors [49]. Understandably, older people with higher education have solid critical thinking skills to screen and capture high-quality information resources. However, less educated older people prefer to rely on visual or oral factors to receive information (i.e., public radio and speeches) [50,51], which leads them to have relatively limited health knowledge from the limited-quality context to motivate them to take DHB.

The results also indicated that older adults living in eastern China took DHB more seriously during the survey period than others reported in the central and western regions. We analyzed the significant differences caused by the multiple SES of different regions. Existing studies have indicated that SES could affect an individual’s health behaviors through various factors such as education, living environment, and receiving health care [46,47,52]. Indeed, in contrast to the prosperous eastern region and the industrial and agricultural central region, the western region, situated inland, is environmentally and economically underdeveloped [53]. Due to the diversity of Chinese geography, there is an imbalance in economic development between the eastern, central, and western regions, which also causes the inequality of accessible health resources and health care services, directly impacting the senior performance of the DHB. 

The result that older adults who lived in high-risk areas possessed less DHB during COVID-19 was not consistent with the previous discussion by Lau et al. [54]—the adults who lived in Hongkong with severe infections tended to seek more frequent healthy behaviors during the severe acute respiratory syndrome (SARS) outbreak in 2003. We analyzed the difference that the different population groups cause. Compared with the general population (aged 18 to 60 years old), the older adults (≥60 years) in this study are more vulnerable to being affected by the living environment. In order to curb the spread of COVID-19, everyone was instructed to “stay at home,” and the strictest “full lockdown” measures were implemented in the high-risk areas, wherein large numbers of confirmed or suspected cases occur [9,55]. The quarantine status may have contributed to the reduction in physical activities and social interactions [30,36], inadequate information about prevention measures [26,56], and the disease-induced fear [18,31], which overwhelmingly adverse effects on the older people’s mental health [31,51,57], and indicated brought inactive performance on DHB [18,31,57].

#### 4.1.2. Health-Related Factors

This study revealed that overweight/obese older people had fewer DHB, with a decreased frequency during the pandemic. A similar trend has been reported by previous studies, which have explored the effects of multiple BMI categories on the performance of DHB in different population groups. For example, Luo L. and Du J. [58] found that adolescents with BMI values higher than 24.0 kg/m^2^ had fewer physical activities; Li Y. et al. [53] observed that seniors with normal weight have better dietary habits, especially with greater awareness of intaking V.F.; and Visser et al. [32] found that older adults with a BMI value of less than 22 kg/m^2^ engage in less healthy eating behaviors, such as eating less prior to the epidemic. The results could be explained by the Theory of Planned Behavior (TPB), suggesting that the normal-weight group with better intentions and beliefs has more positive exercise and healthy eating attitudes and behaviors than the overweight/obese group. The behaviors of the overweight/obese group were explained better by perceived behavioral control [59]. In a study among obese Americans [60], which was the best example, the study population reported understanding the importance of exercise for weight management. However, they did not engage in exercise and had a low intention to do so. In preventing obesity, previous studies have already found that having a healthy lifestyle, including physical activity, V.F. consumption, sleep quality, and avoiding the risk behaviors of drinking alcohol and smoking, are significant in the treatment of being obese [43,59]. In other words, it can be expected that taking up DHB inactivity among overweight/obese older adults will further cause weight gain. In particular, it was previously reported that a higher body mass index was associated with an increased risk for COVID-19 hospitalizations and deaths [11]. With increased cases of weight gain, COVID-19 may have more severe health outcomes in older people, explicitly concerning mortality and disability.

During the survey period, the respondents who reported habits of alcohol consumption also reported a significantly lower frequency of DHB. A similar conclusion was drawn by a literature review [61] and was explored from two studies that indicated the alcohol drinking habit causes a higher rate of physical inactivity [62,63]. In addition to physical activity, previous studies also explored an impacted relationship between alcohol consumption and a healthy diet. From the dataset, China Health and Nutrition Survey (CHNS, 1991–2009), Li Y. et al. [64] found that drinking status was significantly associated with V.F. intake among people aged 60 years and older in China; the subjects were more likely to eat meat and fat-rich items when drinking due to the unique Chinese diet. To our knowledge, the analysis of relationships between alcohol consumption and various DHB categories is limited to a few cases. However, the lesson from the SARS outbreak in 2003 indicated that people who experienced self-isolation or worked in high-risk environments (for example, hospital employees [65]) had higher rates of alcohol abuse and dependence symptoms [54,65,66]. Existing research predicted that the social or interpersonal isolation in the COVID-19 control period would lead to the long-term issue of the public’s daily drinking habits [65,66]. Given the long-term effects of heavy drinking on their health, more health and anti-drinking campaigns are needed, especially for the most at-risk people.

### 4.2. Future Implementations and Suggestions 

The COVID-19 pandemic has provided a powerful contemporary illumination and validation of the need for self-care capabilities and self-care support at population levels. Especially for China, self-care offers a straightforward strategy among the range of measures required to combat COVID-19 and future health threats. In the long run, COVID-19 will not be the only one and the last one of the global pandemics. In short, while looking to the future, self-care should become a new norm in life, which requires the public to possess personal power and practical skills for taking DHB.

In light of the above analysis, the inactivity of DHB affected by the socially disadvantaged, including low education and poor SES, affirm the WHO recommendations for self-care [20] and emphasize the role of government in prompting DHB among older Chinese. Except for the single efforts in health care, the Chinese government is essential in addressing macro-societal factors that adversely affect individuals’ mental and physical health. In particular, the low SES and a lack of resources to meet critical needs and expenses contribute to poor self-care [17]. Thus, the government should focus on and improve resource access, such as housing and food support, and access to health care.

Furthermore, the different sectors of government and healthcare departments should force health education on the older population, which is vital for empowering individuals, families, and communities to optimize their health as advocates for policies that promote and protect health. Specifically, educational efforts should differ depending on the diverse populations. For example, the outreach to seniors with low education should involve brief and easy-understanding information to empower the audience to realize. In addition, overweight seniors face increased risks of lipid disorders, inflammation, and cardiovascular and metabolic disorders [32], which are vulnerable to COVID-19. We suggest paying more attention to the education of overweight or obese older adults, including the potential health threats, the importance of DHB, and practical guidelines for self-care.

To address future threats, only relying on a single effort from administrators and policymakers is not enough [17]; instead, each individual should take responsibility for their health and well-being [20]. Therefore, to achieve the full potential for health, older Chinese citizens should work with health professionals and the government positively to prevent and manage risks to health, wherever and however they arise. For example, we advise alcoholics to enhance adherence to clinical interventions to forester their healthy behaviors. Further, the older population is valued for independence to cope with their illness, and risks threaten their well-being by practicing and enhancing self-care abilities and disease management skills. Notably, older adults are recommended to follow a healthy diet and regular physical activity to sustain an energy balance that prevents weight gain, such as increasing the consumption of healthy foods (for example, fresh fruit and vegetables) and adhering to the recommended physical activities.

### 4.3. Strengths and Limitations

The strengths of the current study include its large sample, the high response rate for the cross-sectional study, and the use of measured DHB to assess the self-care routine of Chinese seniors during COVID-19. However, as with most population-based observational studies, the present study is subject to limitations. First, there was no standardized scale as a reference when designing the questionnaire in this study. Still, the first draft was prepared based on WHO, the official reports of the Chinese Center for Disease Control and Prevention, and related literature, which were finalized after expert evaluation. Second, there may be selection bias. Convenient sampling was used to conduct a questionnaire based on the Internet in this study. Then, the respondents were mostly limited to older adults who were likely to access the Internet. However, it should be noted that China was under strict control measures at the time of this study, and offline research was not possible. A web-based questionnaire may be the most appropriate method of data collection. Third, this study is cross-sectional and cannot show the causal relationship between independent variables and daily health behaviors. Finally, recall bias and the Hawthorne effect may exist.

## 5. Conclusions

The COVID-19 pandemic has caused the public to experience pandemic-related changes in their daily self-care routines. The study found that 1256 older Chinese under each level of variables self-reported their overall DHB performance (including six DHB categories) increased changes compared to the past week. From these, participants who had a higher education status and lived in eastern China reported an arresting increased frequency of taking DHB. However, compared with others, some subgroups reported nonsignificant increasing changes. In particular, older Chinese citizens lived with high-risk infection, were overweight/obese with high BMI values, and had alcohol consumption self-reported changing DHB with an indistinctive increase. We believe self-care is one of the norms that inform human social and political life, underpin human dignity, and support the common good. Thus, these findings can build a foundation for developing healthcare policy and services for the relevant government and departments.

## Figures and Tables

**Table 1:** Univariate analysis of participants’ general information (*socio-demographic characteristics*, *physical well-being*, and *self-assessment of health*) on their DHB performance during the COVID-19 pandemic (*n* = 1256) ^a^.

	Number (%)	DHB (Mean ± SD) ^1^	t/F(df)	*p*-Value ^2^
**Total**	1256 (100)	14.70 ± 2.140	t_(1256)_ = 44.636	**<0.001 ^3^**
**Socio-demographic characteristics**				
Sex			t_(1255)_ = 0.656	0.512
Female	693 (55.2)	14.73 ± 2.152		
Male	563 (44.8)	14.65 ± 2.127		
Age groups (years)			F_(2,1253)_ = 5.786	**0.003**
60~69	616 (49.0)	14.86 ± 2.166 ^d^		
70~79	509 (40.5)	14.62 ± 2.114 ^d^		
≥80	131 (10.4)	14.20 ± 2.043 ^e^		
Marital Status			t_(1255)_ = −3.449	**0.001**
Married/cohabiting	938 (74.7)	14.82 ± 2.143		
Unmarried/divorced/separated/widowed	318 (25.3)	14.34 ± 2.097		
Education level			F_(3,1252)_ = 9.608	**<0.001**
Primary school and below	572 (45.5)	14.39 ± 2.158 ^d^		
Middle School	332 (26.4)	14.76 ± 2.114 ^e^		
High school	197 (15.7)	15.06 ± 2.160 ^e,f^		
College or above	155 (12.3)	15.25 ± 1.919 ^f^		
Residence			t_(1255)_= −3.746	**<0.001**
Rural	677 (53.9)	14.48 ± 2.169		
Urban	579 (46.1)	14.95 ± 2.080		
Retired types ^b^			F_(2,1253)_ = 1.852	0.157
Semi-retirement ^b1^	69 (5.5)	14.67 ± 2.254		
Retirement with honors ^b2^	140 (11.1)	14.37 ± 2.439		
Traditional retirement ^b3^	1047 (83.4)	14.74 ± 2.088		
Monthly household income (RMB) ^b^			F_(2,1253)_ = 4.431	**0.012**
<600	212 (16.9)	14.44 ± 2.279 ^d^		
600~6000	842 (67.0)	14.67 ± 2.138 ^d^		
>6000	202 (16.1)	15.05 ± 1.955 ^e^		
Region of living ^c^			F_(2,1253)_ = 10.887	**<0.001**
Eastern	622 (49.5%)	14.94 ± 2.082 ^d^		
Central	191 (15.2)	14.77 ± 2.140 ^d^		
Western	443 (35.3)	14.33 ± 2.174 ^e^		
COVID-19 risk level (number of infected cases) ^c^			F_(2,1253)_ = 2.666	0.070
Low-risk (<100)	143 (11.4)	14.83 ± 2.043		
Medium-risk (100~999)	594 (47.3)	14.55 ± 2.190		
High-risk (≥1000)	519 (41.3)	14.83 ± 2.102		
**Physical Well-being**			F_(2,1253)_ = 3.848	**0.022**
Classification of BMI (calculated BMI values)				
Underweight (<18.5)	102 (8.1)	14.29 ± 2.319 ^d^		
Normal weight (18.5~23.9)	749 (59.6)	14.82 ± 2.200 ^e^		
Overweight/obesity (≥24.0)	405 (32.2)	14.57 ± 1.960 ^d^		
Smoking			t_(1255)_ = 2.278	**0.023**
No (Never/Have quit smoking)	1018 (81.1)	14.76 ± 2.142		
Yes	238 (18.9)	14.41 ± 2.114		
Drinking			t_(1255)_ = 2.403	**0.016**
No (Never/Have quit drinking)	852 (67.8)	14.79 ± 2.178		
Yes	404 (32.2)	14.49 ± 2.046		
Chronic disease			F_(2,1253)_ = 3.212	**0.041**
No chronic disease	258 (20.5)	14.81 ± 2.098 ^d^		
1–2 chronic disease(s)	800 (63.7)	14.74 ± 2.137 ^d^		
Multiple (3 or more) chronic diseases	198 (15.8)	14.35 ± 2.183 ^e^		
**Self-assessment of health**			F_(3,1252)_ = 2.897	**0.034**
Poor	44 (3.5)	14.43 ± 2.182 ^d,e^		
Fair	561 (44.7)	14.52 ± 2.133 ^d^		
Good	374 (29.8)	14.81 ± 2.118 ^e^		
Very good /Excellent	277 (22.1)	14.93 ± 2.157 ^e^		

Abbreviations: SD, standard deviation; BMI: body mass index; RMB: RenMingBi. ^a^ Of the 1256 participants involved in the current analysis, 722 (57.5%) completed the questionnaire with the help of others (proxy filling). ^b^ The covariates were designed to collect participants’ SES. ^b1^ *Semi-retirement:* the older is leaving his/her chosen career but continuing to work afterward, usually with reduced and flexible hours that let them spend more time enjoying leisure activities [41]. ^b2^ *Retirement with honors*: a unique type in China, which is for the older cadre who are civil servants working in the government, public institutions, and state-owned enterprises, or those who participated in the revolution before the National Day on 1 October 1949 (excluding the day of October 1) [44]. ^b3^ *Traditional retirement*: for all the general older population, who have reached legal age, left their career, and never look back [41]. ^c^ The covariates were designed to collect participants’ living environments during the survey period. ^d,e,f^ There was a common superscript between the different strata groups under each dependent variable, and there was no statistical significance between them (*p* ≥ 0.05). ^1^ Changes in overall DHB performance: the total scores on 6 DHB categories range from 8 to 18 among 1256 participants. ^2^ Statistically significant associated factors are indicated in bold. ^3^ We applied a statistical analysis of one-sample *t*-test.

**Table 2:** Changes on DHB performance among older Chinese in the past week (*n* = 1256).

DHB Categories ^a^	Frequency Changes (Percentage, %) ^b^		
	Decreased	No Change	Increased
Opening the door/window to keep interactive ventilation ^c^	66 (5.3)	243 (19.3)	947 (75.4)
Washing hands ^c^	11 (0.9)	227 (18.1)	1018 (81.1)
Doing physical activities	357 (28.4)	438 (34.9)	461 (36.7)
Eating vegetables and fruits (VF), and rich-protein diets ^c^	62 (4.9)	523 (41.6)	671 (53.4)
Taking vitamins/medical supplements	99 (7.9)	803 (63.9)	354 (28.2)
Having a high-quality sleep with enough hours	64 (5.1)	598 (47.6)	594 (47.3)
Changes in overall DHB (mean ± SD; Range) ^d^	14.70 ± 2.140; (8–18)		

^a^ The particular DHB categories were involved according to the self-care pillars [22] of hygiene (general and personal), nutrition (type and quality of food eaten), lifestyle (sporting activities, leisure, etc.), and self-medication. ^b^ Ordinal variables; grading numerical value of each changing level (Decreased, No change, Increased) as points 1, 2, and 3 to one DHB category, respectively. ^c^ On the basis of ordinal recoding, the median of frequency changes for certain DHB is 3 points (increased). ^d^ Numerical variables; were calculated by adding the valued score of each participants’ self-reported frequency changes on taking six DHB categories week together, ranging from 8 to 18 among 1256 participants.

**Table 3:** Associated factors * impacted DHB among older Chinese during the COVID-19 pandemic (*n* = 1256).

Features	Model 1		Model 2		Model 3	
	β (95% CI)	*p*-Value	β (95% CI)	*p*-Value	β (95% CI)	*p*-Value
Constants	14.250 (13.512, 14.988)	<0.001	14.533 (13.751, 15.315)	<0.001	14.511 (13.481, 15.542)	<0.001
**Block 1: Socio-demographic characteristics**						
Gender						
Female	Raf ^a^		Ref		Ref	
Male	−0.127 (−0.367, 0.113)	0.299	0.017 (−0.251, 0.285)	0.903	0.012 (−0.256, 0.281)	0.928
Age groups (years)						
60~69	Ref		Ref		Ref	
70~79	−0.093 (−0.352, 0.165)	0.478	−0.048 (−0.309, 0.213)	0.718	−0.057 (−0.319, 0.205)	0.669
≥80	**−0.422 (−0.837, −0.006)**	**0.047**	−0.366 (−0.785, 0.054)	0.088	−0.374 (−0.794, 0.046)	0.081
Marital Status						
Unmarried/divorced/separated/widowed	Ref		Ref		Ref	
Married/cohabiting	0.264 (−0.026, 0.555)	0.075	0.231 (−0.059, 0.522)	0.119	0.233 (−0.058, 0.524)	0.116
Education level						
Primary school and below	Ref		Ref		Ref	
Middle School	0.221 (−0.087, 0.529)	0.159	0.184 (−0.124, 0.492)	0.241	0.161 (−0.150, 0.471)	0.310
High school	**0.461 (0.070, 0.853)**	**0.021**	**0.429 (0.037, 0.820)**	**0.032**	**0.403 (0.009, 0.797)**	**0.045**
College or above	**0.573 (0.122, 1.024)**	**0.013**	**0.505 (0.052, 0.958)**	**0.029**	**0.488 (0.034, 0.943)**	**0.035**
Residence						
Rural	Ref		Ref		Ref	
Urban	0.066 (−0.223, 0.354)	0.656	0.059 (−0.231, 0.349)	0.689	0.066 (−0.224, 0.357)	0.655
Retired types						
Semi-retirement	Ref		Ref		Ref	
Retirement with honors	0.030 (−0.591, 0.651)	0.924	−0.042 (−0.666, 0.582)	0.895	0.008 (−0.621, 0.637)	0.980
Traditional retirement	0.208 (−0.316, 0.732)	0.436	0.141 (−0.388, 0.669)	0.601	0.179 (−0.353, 0.711)	0.509
Monthly household income (RMB)						
<600	Ref		Ref		Ref	
600–6000	−0.059 (−0.396, 0.279)	0.733	−0.052 (−0.390, 0.287)	0.765	−0.058 (−0.398, 0.282)	0.739
>6000	0.092 (−0.367, 0.552)	0.694	0.098 (−0.363, 0.558)	0.677	0.062 (−0.403, 0.526)	0.795
Region of living						
Western	Ref		Ref		Ref	
Eastern	**0.730 (0.354, 1.106)**	**<0.001**	**0.791 (0.413, 1.169)**	**<0.001**	**0.771 (0.392, 1.151)**	**<0.001**
Central	**0.395 (0.012, 0.778)**	**0.043**	**0.388 (0.004, 0.772)**	**0.048**	0.373 (−0.012, 0.759)	0.057
COVID-19 risk level (number of infected cases)						
Low-risk (<100)	Ref		Ref		Ref	
Medium-risk (100~999)	−0.287 (−0.706, 0.132)	0.179	−0.363 (−0.785, 0.059)	0.091	−0.346 (−0.770, 0.077)	0.109
High-risk (≥1000)	**−0.685 (−1.187, −0.183)**	**0.008**	**−0.779 (−1.283, −0.274)**	**0.003**	**−0.740 (−1.248, −0.231)**	**0.004**
**Block 2: Physical well-being**						
Classification of BMI (calculated BMI values)						
Underweight (<18.5)	- ^b^	-	Ref		Ref	
Normal weight (18.5~23.9)	-	-	−0.420 (−0.863, 0.023)	0.063	−0.424 (−0.869, 0.020)	0.061
Overweight/obesity (≥24.0)	-	-	**−0.264 (−0.524, −0.003)**	**0.048**	**−0.265 (−0.526, −0.004)**	**0.047**
Smoking						
No (Never/Have quit smoking)	-	-	Ref		Ref	
Yes	-	-	−0.050 (−0.395, 0.295)	0.776	−0.040 (−0.385, 0.306)	0.822
Drinking						
No (Never/Have quit drinking)	-	-	Ref		Ref	
Yes	-	-	**−0.341(−0.624, −0.057)**	**0.019**	**−0.350 (−0.634, −0.065)**	**0.016**
Chronic disease						
No chronic disease	-	-	Ref		Ref	
1–2 chronic disease(s)	-	-	0.075 (−0.225, 0.375)	0.625	0.139 (−0.179, 0.457)	0.391
Multiple (3 or more) chronic diseases	-	-	−0.166 (−0.576, 0.245)	0.429	−0.073 (−0.515, 0.369)	0.745
**Block 3: Self-assessment of health**						
Poor	-	-	-	-	Ref	
Fair	-	-	-	-	−0.152 (−0.807, 0.504)	0.650
Good	-	-	-	-	−0.016 (−0.697, 0.665)	0.964
Very good/Excellent	-	-	-	-	0.068 (−0.640, 0.777)	0.850

Abbreviations: 95% CI: 95% confidence interval; BMI, body mass index; RMB: RenMingBi. * Statistically significant associations are indicated in bold. ^a^ Ref: reference group; ^b^: Data was not involved in the model.

## Data Availability

The collected data files are available on request from the corresponding author, Hongmei Wang: rosa@zju.edu.cn.

## Supplementary Materials

The following supporting information can be downloaded at: https://www.mdpi.com/article/10.3390/nu14081678/s1, Table S1: Statistical measures of the three models under multivariable analysis (*p* < 0.001 for all models).
