# Supplementary material for: Life in a New Normal with a Self-Care Routine: A Cross-Sectional Study of Older Adults’ Daily Health Behaviors (DHB) Performance during the Initial Outbreak of COVID-19 in China
Source: Nutrients. 2022 Apr 18;14(8):1678. doi: 10.3390/nu14081678 (PMC9024498; doi:10.3390/nu14081678)
Supplement: Supplementary file 1 [file nutrients-14-01678-s001.zip › nutrients-1630237-supplementary.pdf]

Supplementary Table S1. Statistical measures of the three models under multivariable analysis (*p* < 0.001 for all models)

Measures	Model 1	Model 2	Model 3
F	3.808	3.453	3.111
R^2	0.047	0.058	0.059
$\triangle R^2$	0.047	0.011	0.001
Adjusted R^2	0.035	0.041	0.040
